# Epidemiology of Diabetic Foot Ulcers and Amputations in Romania: Results of a Cross-Sectional Quality of Life Questionnaire Based Survey

**DOI:** 10.1155/2016/5439521

**Published:** 2016-02-25

**Authors:** Cosmina I. Bondor, Ioan A. Veresiu, Bogdan Florea, Etta J. Vinik, Aaron I. Vinik, Norina A. Gavan

**Affiliations:** ^1^Department of Medical Informatics and Biostatistics, Iuliu Hațieganu University of Medicine and Pharmacy, 6 Pasteur Street, 400349 Cluj-Napoca, Romania; ^2^Department of Diabetes, Nutrition and Metabolic Diseases, Iuliu Hațieganu University of Medicine and Pharmacy, 4-6 Clinicilor Street, 400006 Cluj-Napoca, Romania; ^3^IMOGEN Research Center, Iuliu Hațieganu University of Medicine and Pharmacy, 8 Victor Babeș Street, 400012 Cluj-Napoca, Romania; ^4^Eastern Virginia Medical School, Strelitz Diabetes Center, 855 West Brambleton Avenue, Norfolk, VA 23510, USA; ^5^Research & Neuroendocrine Unit, Eastern Virginia Medical School, 855 West Brambleton Avenue, Norfolk, VA 23510, USA; ^6^Society of Diabetic Neuropathy, Wörwag Pharma GmbH & Co. KG, Romanian Representative Office, 11 Fagului Street, 400483 Cluj-Napoca, Romania

## Abstract

This is a post hoc analysis of quality of life in diabetic neuropathy patients in a cross-sectional survey performed in 2012 in Romania, using the Norfolk QOL-DN in which 21,756 patients with self-reported diabetes were enrolled. This current analysis aims to expand research on the diabetic foot and to provide an update on the number of foot ulcers found in Romania. Of the 21,174 patients included in this analysis, 14.85% reported a history of foot ulcers and 3.60% reported an amputation. The percentage of neuropathy patients with foot ulcers increased with age; the lowest percentage was observed in the 20–29-year age group (6.62%) and the highest in the 80–89-year age group (17.68%). The highest number of amputations was reported in the 70–79-year age group (largest group). Compared to patients without foot ulcers, those with foot ulcers had significantly higher scores for total DN and all its subdomains translating to worse QOL (*p* < 0.001). This analysis showed a high rate of foot ulcers and amputations in Romanian diabetic patients. It underscores the need for implementation of effective screening and educational programs.

## 1. Introduction

Diabetes represents a major risk factor for lower limb amputations; it has been estimated that the presence of diabetes is associated with a 20-fold higher risk of lower limb amputations as compared to people without diabetes [1]. Diabetes-related foot ulcers have been reported with an annual incidence of 2% and a lifetime risk of 25% and are considered a major cause of nontraumatic lower extremity amputations [2]. Additionally, it has been shown that these complications have a major impact on the quality of life (QOL) and psychological status of diabetic patients [3, 4] and, as a consequence, the patients' QOL has been recognized as a measure of treatment effect [5].

Due to increased healthcare resources utilization [6] and work-loss associated costs, diabetic foot ulcers and amputations represent a major burden for the healthcare systems in both developed and developing countries. According to a health economic analysis performed in the USA, the diabetic foot ulcers are associated with $9 billion to $13 billion increase in the direct yearly costs, thus doubling the costs of diabetes care [6]. In Romania, extrapolating the results of local studies (unpublished data), we have estimated an annual direct expenditure only for lower extremity amputations in patients with diabetes, of around 2.5 mil EUR. In the context of increasing incidence and prevalence of diabetes, a decrease in the prevalence of ulcers and lower limb amputations cannot be expected without specifically designed population interventions.

Limited data on epidemiology of diabetic foot ulcers and lower limb amputations are available for Romania [7, 8]. A research study performed in 2003, including data from several diabetes clinics from Romania, reported that the prevalence of foot ulcers was 3.2% in patients with type 1 diabetes and 3.8% in patients with type 2 diabetes [7]. Recently, an analysis of the number of lower limb amputations in patients with diabetes showed an increasing trend between 2006 and 2010 [8]. This increase was attributable to a dramatic increase in the rates of amputations in persons with type 2 diabetes as compared to 2006; since then, the number of amputations in this population increased with 16.96% in 2007, 60.75% in 2008, 66.91% in 2009, and 104.64% in 2010 [8]. To the best of our knowledge, no additional data are available on the incidence or prevalence of diabetes foot ulcers for this population. However, it is known that the incidence of lower extremity amputations is a marker of the quality of diabetic foot disease management [9, 10], with high amputation possibly attributable to inadequate education of patients and late presentation or inadequate resources for the management of the diabetic foot [11].

The analysis presented here aims to expand the research on the status of the diabetic foot in Romania and to provide an up-to-date status on the frequency of foot ulcers. This is a post hoc analysis of the Quality of Life in Patients with Diabetic Neuropathy in Romania Study (QOL-DN Romania), which had the main objective to assess the prevalence of self-reported diabetic neuropathy in Romanian population and its impact on the QOL by using the Norfolk QOL-DN “fiber-specific” questionnaire, professionally translated to Romanian. It was a cross-sectional survey performed in 2012 which enrolled 21,756 patients with diabetes and showed prevalence of neuropathy of 79% in this population [12].

## 2. Materials and Methods

### 2.1. Protocol and Survey Population

This was a cross-sectional survey in which 25,000 Romanian-translated Norfolk QOL-DN questionnaires were distributed by 181 Romanian healthcare providers (153 physicians (diabetes specialists), 5 neurologists, 14 general practitioners, and 9 nurses) to their patients with diabetes between January and December 2012. The Romanian version of the Norfolk QOL-DN is a self-administered questionnaire comprised of 16 items that capture demographic and medical history data (not scored) and 35 scored items related to patients' perception of their own health signs, symptoms, and the impact of diabetic neuropathy on their daily life over the previous 4 weeks. For the analysis of the nonscored items, we included age and the responses to the following questions: “Do you have diabetes?,” “Do you have neuropathy?,” “Have you ever had ulcers on your feet?,” and “Have you ever had any amputation?” Total QOL and subdomain (physical functioning/large-fiber neuropathy, symptoms, activities of daily living (ADLs), autonomic neuropathy, and small-fiber neuropathy) scores were calculated based on responses to the scored items, with higher scores corresponding to poorer QOL. The survey design, the survey population, and a detailed description of the Romanian version of the Norfolk QOL-DN were previously reported [12].

All patients were informed that their personal data would be analyzed as part of a survey registered with the Romanian authorities and consented for their data to be included in the analysis. The survey was approved by the National Supervisory Authority for Personal Data Processing under number 0006753.22-03-2012.

### 2.2. Statistical Analysis

Frequency tables, contingency tables, and graphics were used for the description of the qualitative variables. The total QOL scores and the scores for each subdomain are presented as mean ± standard error (SE) and were compared using the Mann-Whitney test. The age is presented as mean ± standard deviation. For the variables presented as percentages, we tested the significance of differences by one-way analysis of variance, the Scheffé post hoc test, and the Chi-square test.

All descriptive and inferential analyses were performed using IBM SPSS Statistics for Windows, Version 15.0 (Armonk, NY: IBM Corp.). The significance threshold was *α* = 0.05.

## 3. Results and Discussions

### 3.1. Results

**Figure 1:**
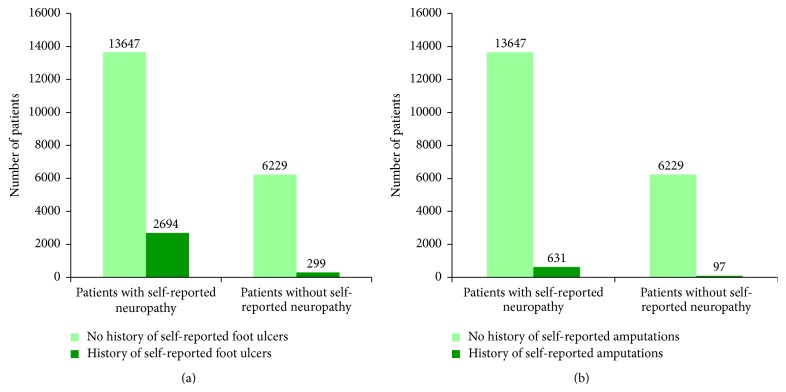
Numbers of self-reported foot ulcers (a) and amputations (b) in the population included in the analysis stratified by self-reported neuropathy.

As previously described [12], of the 25,000 questionnaires distributed, 23,543 were returned. Of these, after removing those not valid, with missing answer or “No” as an answer to the question “Do you have diabetes?,” 21,174 were included in the present analysis. Of these, 13,812 patients answered “Yes” to the question “Do you have neuropathy?” and 7362 answered “No” to the same question. A history of foot ulcers was reported by 3088 (14.66%) patients with self-reported diabetes. The frequency of both foot ulcers and amputations was significantly higher among patients who self-reported neuropathy compared to those who did not self-report neuropathy (2,694/13,812 (19.50%) versus 299/6229 (4.8%) patients with a history of foot ulcers and 638/13,812 (4.62%) versus 89/7,362 (1.21%) patients with a history of amputation, resp.). Of the patients who answered “Yes” to the question “Do you have diabetes?,” 750 (3.5%) reported that they had an amputation (Figure 1). Mean age was similar in the total group that reported diabetes irrespective of the history of neuropathy and foot ulcers, the group that reported neuropathy, the one that reported foot ulcers, and the one that reported amputations: 60.87 ± 11.31 years, 61.73 ± 10.99 years, 62.12 ± 11.06 years, and 62.44 ± 10.91 years, respectively.

The percentage of patients with neuropathy increased with age, from 39.34% in the 20–29-year age group to 76.91% in the 80–89-year age group. A similar trend was observed for the percentage of patients with foot ulcers among those with neuropathy; the lowest percentage was observed in the 20–29-year age group (6.62%) and the highest in the 80–89-year age group (17.68%). The highest frequency of amputations was reported in the 70–79-year age group (15.03%) (Table 1).

**Figure 2:**
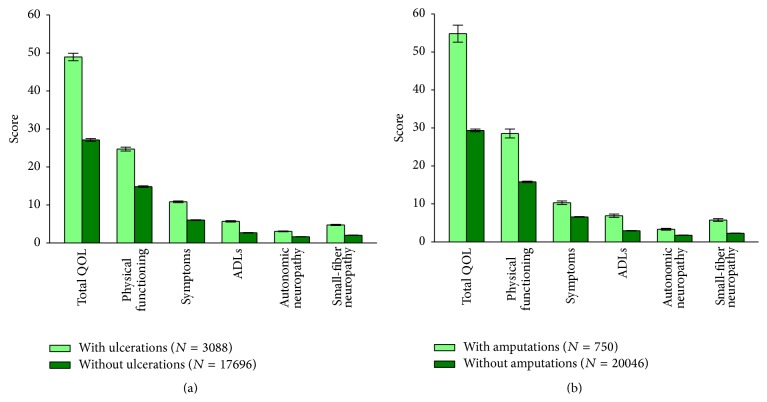
Norfolk QOL-DN total and subscale scores in Romanian patients with self-reported diabetes mellitus with and without foot ulcers (a) and with and without amputations (b). QOL: quality of life; ADLs: activities of daily living; *N*: number of patients in a given category.

In the whole analyzed group, the mean scores for total QOL, symptoms, ADLs, autonomic neuropathy, physical functioning/large-fiber neuropathy, and small-fiber neuropathy in those with and without foot ulcers and amputations are presented in Figure 2. Compared to patients without foot ulcers, those with foot ulcers had significantly higher scores for total QOL and all subdomains: 48.95 versus 27.12 for total QOL; 10.83 versus 6.01 for symptoms; 5.66 versus 2.66 for ADLs; 3.04 versus 1.62 for autonomic neuropathy; 4.72 versus 2.01 for small-fiber neuropathy; and 24.70 versus 14.82 for physical functioning/large-fiber neuropathy subdomain (*p* < 0.001). Similar differences were observed between those with amputations and those without amputations: 54.83 versus 29.36 for total QOL score; 10.31 versus 6.58 for symptoms; 6.89 versus 2.94 for ADL; 3.33 versus 1.76 for autonomic neuropathy; 5.76 versus 2.28 for small-fiber neuropathy; and 28.54 versus 15.79 for physical functioning subdomain (*p* < 0.001).

When data were compared according to the presence of neuropathy and foot ulcers, patients with neuropathy and a history of foot ulcers had the highest scores for total QOL and all subdomain scores: 51.53 for total QOL; 11.45 for symptoms; 6.00 for ADLs; 5.02 for small-fiber neuropathy; 3.21 for autonomic neuropathy; and 25.85 for physical functioning/large-fiber neuropathy subdomain. The presence of neuropathy with or without foot ulcers was associated with higher total QOL and subdomain scores as compared to the ones in patients with foot ulcers but without self-reported neuropathy. The lowest scores were reported in those with no self-reported neuropathy and no history of foot ulcers (Table 2).

**Figure 3:**
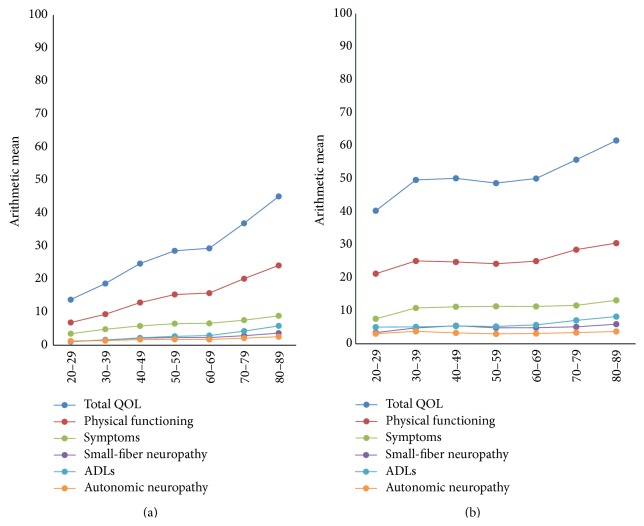
Norfolk QOL-DN total and subscale scores by groups of age in patients with self-reported diabetes mellitus (*n* = 21,174; *p* < 0.001, (a)) and diabetes, neuropathy, and foot ulcers (*n* = 815; only ADLs, *p* < 0.05, (b)).

Stratifying the total QOL scores on age decades (Figure 3), we observed that the QOL of a patient aged 20–29 years who reported previous foot ulcers is similar to the QOL of a patient aged 80 years with self-reported diabetes with or without neuropathy or foot ulcers.

### 3.2. Discussion

The analysis of this sample population of Romanian diabetic patients showed a high frequency of history of foot ulcers (14.6%). The frequency was significantly higher among patients who self-reported neuropathy compared to those who did not self-report neuropathy (19.50% versus 4.06%) and increased with age from 6.62% in those aged 20–29 years to 17.68% in those aged 80–89 years. Our results are in line with previously reported data from the US that showed that the lifetime risk for a diabetic patient to experience a foot ulcer is 15 to 25% [13]. However, lower rates were previously reported in Europe; two cross-sectional community surveys performed in the UK showed that 5.3% to 7.4% of patients with diabetes had a history of foot ulcer [14, 15]. A study performed in Greece reported a rate of foot ulcers of 4.75% (95% confidence limits: 3.3%–6.2%) [16]; in a community in Sweden, 10% of the population included in the analysis had a history of foot ulcers and an additional 2% reported having present ulcers [17].

Although peripheral diabetic neuropathy is currently recognized as the leading risk factor for the development of the foot ulcers, it has been shown that the presence of this complication* per se* is not sufficient for the development of foot ulcers. Reiber et al. [18] showed in a clinical study that peripheral neuropathy was present in 78% of the patients who developed a foot ulcer, while foot deformities were present in 63% and peripheral vascular disease in 35% of the patients. We have not evaluated the frequency of peripheral arterial disease and of the neuropathy-associated foot deformities and therefore we cannot exclude the coexistence of these conditions in patients with self-reported neuropathy and a history of foot ulcers. Additionally, in patients without self-reported neuropathy, due to the lack of the objective evaluation of the neuropathy, we cannot claim that the foot ulcers were due to the peripheral artery disease in all cases.

In our survey, 3.50% of the patients reported a history of amputation; the frequency was significantly higher in the group with self-reported neuropathy compared to the one without (4.62% versus 1.21%). The majority of the information on the incidence and prevalence of the lower limb amputation is originating from hospital discharge data; therefore, a comparison with the reported data is difficult. In the UK and Spain, the incidence of the lower limb amputations was reported as ranging from 5.8 to 31 per 10^5^ patients/year [19–21]. For Romania, the recently reported mean crude incidence of lower limb amputations in patients with diabetes is 21.3 per 10^5^ [8]. Between 2006 and 2010, an increase in the lower limb amputation rates was observed for the patients with type 1 and type 2 diabetes combined, but the overall increase was due to an increase in the incidence of type 2 diabetes, while the rates in type 1 diabetes decreased [8]. This observation is in line with data from other European countries, all reporting a decrease in the incidence of the amputations in type 1 diabetes [21–23] and an increase in the amputations in type 2 diabetes [21, 22].

In our analysis, we observed an important frequency of foot ulcers and amputations in the active population aged 20–60 years. In these age groups, the frequency of foot ulcers and amputations was 6.62% and 0.74% in those aged 20–29 years and increased in parallel with age, reaching 12.27% and 2.88% in those aged 50–59 years. These results are similar to data published by Veresiu et al. [8], which showed an increase in the amputation rates for both type 1 and type 2 diabetes in persons aged 30–39 years from 2006 until 2010. The authors of this study reported a decrease in the incidence of lower limb amputations in the 20–29-year age group, while for the other age groups, the incidence increased for patients with type 2 diabetes and decreased for those with type 1 diabetes [8]. Our observations and the ones deriving from the data on the incidence of lower limb amputations are of special concern. Despite improvements in the standard of care of the diabetic foot in Romania, ulcers and amputations are being reported at young ages in the active population and are associated with higher direct and indirect costs. A possible explanation for this observation might be the variation in the delivery of preventive measures and foot care in patients at the local level; currently, physicians specialized in diabetic foot care are available only in large hospitals and large cities [8]. This may have led to limited access to specific education for an important part of the diabetic patients, with consequences on the level of health literacy, understanding and implementing preventive measures, and referral to the physician in early stages of the pathology.

To further evaluate the impact of the ulcers and amputations, we evaluated the QOL of these patients. The concomitant presence of neuropathy and ulcers or of neuropathy and amputations had a higher impact on the QOL than the presence of each of these alone. Additionally, the presence of neuropathy alone (without a history of foot ulcers) had a higher impact on the QOL than the history of foot ulcers without self-reported neuropathy. It is accepted that patients with neuropathy and those with neuropathy and chronic foot ulcers or amputations have lower QOL compared to their diabetic fellows without neuropathy [3, 4, 24].

We acknowledge that our analysis is based on patients' self-reported information and therefore has all the limitations of such kinds of studies. The most important one is the recall bias which can influence patient's ability to correctly report previous diagnoses. At the same time, it is important to mention that the Norfolk QOL questionnaire used is a rigorously validated instrument that has the ability to discriminate between patients with and without diabetic neuropathy and between different stages of neuropathy severity [25, 26]. A recent systematic review of disease-specific measurement instruments for health-related QOL in diabetic neuropathy [27] concluded based on the evidence for test-retest reliability and known groups validity that the Norfolk QOL-DN is an instrument with the most robust psychometric properties in treatment evaluation. Whether this assumption is also valid for Norfolk QOL-DN as an instrument for epidemiological data collection remains to be investigated in future prospective studies.

## 4. Conclusion

This analysis showed a high frequency of foot ulcers and amputations in Romanian diabetic patients. The relative high frequency of these among the younger age groups is of special concern. This analysis offers an overview of the diabetic foot problems and underlines the need for planning and implementation of effective screening and educational programs and also the need of increasing the access of diabetic patients to healthcare providers specialized in foot care.

## Figures and Tables

**Table 1:** Patient distribution by age.

Age (years)		20–29	30–39	40–49	50–59	60–69	70–79	80–89
Total group (self-reported diabetes) with available data on age	*n*	272	555	1897	6413	7219	4000	758

Diabetes with neuropathy	*n*	107	310	1111	4127	4721	2817	583
	%	39.34	55.86	58.56	64.66	65.40	70.43	76.91

Diabetes, neuropathy with diabetic foot ulcers	*n*	18	44	212	787	886	601	134
	%	6.62	7.93	11.18	12.27	12.81	15.03	17.68

Diabetes, neuropathy with amputation	*n*	2	9	52	185	213	137	33
	%	0.74	1.62	2.74	2.88	2.95	33.43	4.35

*n*: number of patients in a given category; %: percentage.

Note: no additional data on amputation (i.e., major or minor) was collected.

**Table 2:** QOL differences in age groups shown in Norfolk QOL-DN total and subscale scores (mean ± standard error) in patients with self-reported diabetes mellitus according to the presence of self-reported neuropathy and foot ulcers.

	Self-reported neuropathy, no foot ulcers	Self-reported neuropathy, history of foot ulcers	No self-reported neuropathy, history of foot ulcers	No self-reported neuropathy, no foot ulcers
	*N* = 10953	*N* = 2694	*N* = 299	*N* = 6130
Total QOL	35.18 ± 0.24	51.53 ± 0.53	28.86 ± 1.42	12.91 ± 0.23
Physical functioning	18.96 ± 0.13	25.85 ± 0.28	15.87 ± 0.80	7.51 ± 0.14
Symptoms	7.93 ± 0.05	11.45 ± 0.12	6.08 ± 0.31	2.65 ± 0.046
Small-fiber neuropathy	2.73 ± 0.03	5.02 ± 0.08	2.14 ± 0.18	0.75 ± 0.03
ADLs	3.55 ± 0.04	6.00 ± 0.10	2.93 ± 0.26	1.09 ± 0.04
Autonomic neuropathy	2.01 ± 0.02	3.21 ± 0.06	1.83 ± 0.13	0.91 ± 0.02

QOL: quality of life; ADLs: activities of daily living.
